# MicroRNAs in Renal Cell Carcinoma: Diagnostic Implications of Serum miR-1233 Levels

**DOI:** 10.1371/journal.pone.0025787

**Published:** 2011-09-30

**Authors:** Lena M. Wulfken, Rudolf Moritz, Carsten Ohlmann, Stefan Holdenrieder, Volker Jung, Frank Becker, Edwin Herrmann, Gisela Walgenbach-Brünagel, Alexander von Ruecker, Stefan C. Müller, Jörg Ellinger

**Affiliations:** 1 Klinik und Poliklinik für Urologie und Kinderurologie, Universitätsklinikum Bonn, Bonn, Germany; 2 Klinik und Poliklinik für Urologie, Universitätsklinikum Münster, Münster, Germany; 3 Klinik und Poliklinik für Urologie, Universitätsklinikum des Saarlandes, Homburg/Saar, Germany; 4 Institut für Klinische Chemie und Klinische Pharmakologie, Universitätsklinikum Bonn, Bonn, Germany; 5 Urologische Gemeinschaftspraxis und Ambulatorium Derouet/Poenicke/Becker, Neunkirchen, Germany; 6 Institut für Pathologie, Universitätsklinikum Bonn, Bonn, Germany; Barts & The London School of Medicine and Dentistry, Queen Mary University of London, United Kingdom

## Abstract

**Background:**

MicroRNA expression is altered in cancer cells, and microRNAs could serve as diagnostic/prognostic biomarker for cancer patients. Our study was designed to analyze circulating serum microRNAs in patients with renal cell carcinoma (RCC).

**Methodology/Principal Findings:**

We first explored microRNA expression profiles in tissue and serum using TaqMan Low Density Arrays in each six malignant and benign samples: Although 109 microRNAs were circulating at higher levels in cancer patients' serum, we identified only 36 microRNAs with up-regulation in RCC tissue and serum of RCC patients. Seven candidate microRNAs were selected for verification based on the finding of up-regulation in serum and tissue of RCC patients: miR-7-1*, miR-93, miR-106b*, miR-210, miR-320b, miR-1233 and miR-1290 levels in serum of healthy controls (n = 30) and RCC (n = 33) patients were determined using quantitative real-time PCR (TaqMan MicroRNA Assays). miR-1233 was increased in RCC patients, and thus validated in a multicentre cohort of 84 RCC patients and 93 healthy controls using quantitative real-time PCR (sensitivity 77.4%, specificity 37.6%, AUC 0.588). We also studied 13 samples of patients with angiomyolipoma or oncocytoma, whose serum miR-1233 levels were similar to RCC patients. Circulating microRNAs were not correlated with clinical-pathological parameters.

**Conclusions/Significance:**

MicroRNA levels are distinctly increased in cancer patients, although only a small subset of circulating microRNAs has a tumor-specific origin. We identify circulating miR-1233 as a potential biomarker for RCC patients. Larger-scaled studies are warranted to fully explore the role of circulating microRNAs in RCC.

## Introduction

Kidney cancer is one of the most common malignancies in developed countries [1]. Renal tumors are asymptomatic and non-palpable in early stages; more than 50% of renal tumors are detected incidentally by imaging to investigate non-specific symptoms. The most common renal tumor is clear cell renal cell carcinoma (ccRCC), but other histological subtypes of renal cell carcinoma (i.e. papillary, pRCC; or chromophobe RCC, chRCC) or non-malignant renal tumors (i.e. oncocytoma and angiomyolipoma) are more or less difficult to differentiate by imaging modalities, and additional diagnostic tools are warranted to optimize the management of patients with renal tumors. So far, non-invasive biomarkers are not used in the routine practice because they do not improve the diagnostic or prognostic accuracy [2].

MicroRNAs are small non-coding RNAs of approximately 22 nt size, and modulate differentiation, growth, apoptosis and proliferation of cells. MicroRNA expression profiles not only allow distinguishing malignant and non-malignant tissue, but also distinguishing different tumor entities.[3] MicroRNAs are circulating in a cell-free form in blood [4]–[6], most probably in exosomes which protect them against degradation by RNase [4], [5]. MicroRNA signatures in blood are similar in men and women, as well as individuals of different age.[6] Furthermore, microRNA levels are similar in plasma and serum [4], and freeze/thaw as well as prolonged storage at room temperature do not affect microRNA levels [4]. Thus, circulating microRNAs have the potential of a novel biomarker.

Several studies demonstrated that specific microRNAs are useful to distinguish cancer patients and healthy controls: e.g., patients with prostate cancer have increased levels of miR-26a [7], miR-29 and miR-92a were increased in colon cancer patients [8], and miR-195 was increased in patients with breast cancer [9]. In addition, microRNAs were prognostic indicators (prostate cancer: miR-141 [10]; colon cancer: miR-29 [8]).

## Methods

### Objectives

**Figure 1:**
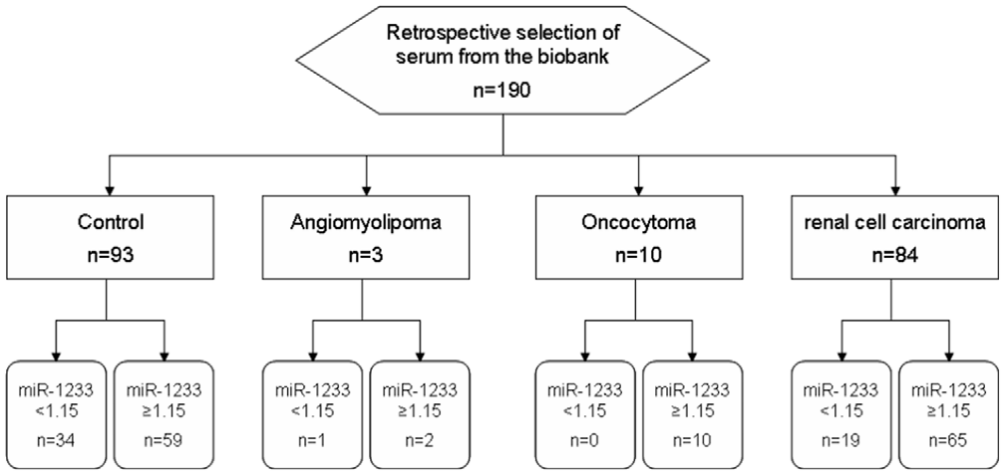
Flow Chart: Verification of miR-1233 as diagnostic marker of renal cell carcinoma.

So far, circulating microRNAs have not been investigated in patients with renal cell carcinoma. Several groups analyzed microRNA expression in tissue, with conflicting results. We therefore designed our study to identify potential candidates using an array technology (TaqMan Low Density Array), and validate the most interesting microRNAs in an independent cohort using conventional real-time PCR. The flow chart of the marker verification study is shown in Figure 1.

### Participants

We collected prospectively serum from patients undergoing radical nephrectomy or nephron-sparing surgery for renal tumors; thus, the study cohort consisted of patients with renal cell carcinoma and benign renal tumors (BRT; i.e. oncocytoma and angiomyolipoma). We also investigated a control group consisting of patients with non-malignant disease (men/women attending to our hospital for surgery of non-malignant disease, i.e. benign prostate enlargement or urinary incontinence, or preventive medical examination). The detailed clinical-pathological parameters of patients are reported in Table 1. The collection of serum samples was performed between 2005 and 2011 at the Departments of Urology at the Universitätsklinikum Bonn (UKB), the Universitätsklinikum Münster (UKM) and the Universitätsklinikum des Saarlandes (UKS).

**Table 1:** Clinical-pathological parameters.

	PHASE I		PHASE II		PHASE III		
	RCC	HI	RCC	HI	RCC	HI	BRT
**Sex**							
male	3	3	27	21	56	68	6
female	3	3	6	9	28	25	7
**Age**							
mean	66.0	47.7	62.7	60.2	60.9	63.7	59.5
median	66.0	47.5	65.0	63.0	63.0	64.0	63.0
range	57–75	40–60	43–80	26–85	25–82	28–88	21–84
**Histology**							
ccRCC	6	n.a.	33	n.a.	69	n.a.	n.a.
pRCC	0	n.a.	0	n.a.	10	n.a.	n.a.
chRCC	0	n.a.	0	n.a.	3	n.a.	n.a.
sRCC	0	n.a.	0	n.a.	2	n.a.	n.a.
**Pathological stage**							
pT1	2	n.a.	23	n.a.	55	n.a.	n.a.
pT2	1	n.a.	3	n.a.	4	n.a.	n.a.
pT3	3	n.a.	7	n.a.	23	n.a.	n.a.
pT4	0	n.a.	0	n.a.	2	n.a.	n.a.
vascular invasion	3	n.a.	3	n.a.	12	n.a.	n.a.
lymph node metastasis	0	n.a.	0	n.a.	8	n.a.	n.a.
distant metastasis	0	n.a.	0	n.a.	5	n.a.	n.a.
**Fuhrman Grade**							
G1	0	n.a.	4	n.a.	11	n.a.	n.a.
G2	6	n.a.	28	n.a.	62	n.a.	n.a.
G3	0	n.a.	1	n.a.	8	n.a.	n.a.
G4	0	n.a.	0	n.a.	3	n.a.	n.a.

*Abbreviations*: RCC, renal cell carcinoma; HI, healthy individuals; BRT, benigne renal tumor; ccRCC, clear cell RCC; pRCC, papillary RCC; chRCC, chromophobe RCC; sRCC, sarcomatoid RCC; n.a., not applicable.

Blood samples were obtained prior surgery. Blood was withdrawn in serum S-Monovette Gel tubes with clotting activator (Sarstedt, Nümbrecht, Germany); after clotting, serum was separated after centrifugation (10 min, 2800 g) and stored in cryotubes at −80°C. All samples were processed between 1 and 3 hours. We also analyzed formalin-fixed, paraffin embedded tissue from six patients stored in the archival files of the Institut für Pathologie at the Universitätsklinikum Bonn.

### RNA isolation

Serum RNA isolation was performed as published earlier [7]. In brief, the mirVana PARIS Kit (Ambion, Foster City, CA, USA) was used to purify RNA from 400 µl serum. RNA isolation was performed according to the manufacturers' recommendation (final elution volume 50 µl) with one exception: we added 25 fmol of a synthetic Caenorhabditis elegans microRNA, cel-miR-39 (Qiagen, Hilden, Germany; catalog number MSY0000010) to the serum before starting the isolation procedure. Quantification of cel-miR-39 allows controlling sample-to-sample differences of RNA isolation efficiency. We analyzed separate (and not pooled) samples at each step of the study [phase-1: ccRCC n = 6, healthy control n = 6; phase-2: ccRCC n = 33, healthy control n = 30; phase-3: RCC n = 84 (including 69 ccRCC, 10 pRCC, 3 chRCC and 2 sRCC patients), healthy control n = 93, angiomyolipoma n = 3, oncocytoma n = 10].

Malignant and non-malignant renal tissue was marked on haematoxylin and eosin stained slides by a pathologist. Five serial sections (20 µm) were cut from the tissue block, and the tissue was dissected using a scalpel to enrich ccRCC and adjacent non-malignant renal tissue. Afterwards, the RNA was isolated with the RecoverAll Total Nucleic Acid Isolation Kit (Ambion) according the manufacturer recommendations. RNA quantity and purity was determined using the NanoDrop 1000 spectrophotometer (ThermoScientific, Wilmington, DE; USA).

### Low Density Arrays

Reverse transcription was performed using the TaqMan MicroRNA Reverse Transcription Kit and Megaplex RT Primers. The reaction mix included 16.6 ng/ml RNA (tissue) or 3 µl of the eluted RNA (serum). In order to increase the sensitivity of the TaqMan Low Density Arrays, we performed a pre-amplification using the TaqMan PreAmp Mastermix. MicroRNA profiling of 754 different human microRNAs was then performed using the TaqMan Low Density Array (TaqMan Array Human MicroRNA A+B Cards Set v3.0; see Table S1 for a list of microRNAs) on an ABIPrism 7900HT. In order to normalize serum microRNA levels, we additional determined miR-39 levels: RNA was reverse transcribed with miR-39 RT primers using the TaqMan MicroRNA Reverse Transcription Kit and quantified using a real-time PCR (see below for details). All reactions were performed as specified in the protocols of the manufacturer (Applied Biosystems, Foster City, CA, USA). Details are reported MIQE-compliant in Methods S1.

### Quantitative Real-Time PCR

We identified seven candidates using the Low Density Array technology for further characterization. The determination of microRNA levels in subsequent validation experiments was performed similar as described above with one exception: Reverse Transcription was performed with a self-created primer pool: To obtain this RT primer pool, we speed vacuumed a mixture of each 320 µl of cel-miR-39 (Applied Biosystems Assay ID: 000200), hsa-miR-1233 (ID: 002768), hsa-miR-106b* (ID: 003280), hsa-miR-1290 (ID: 002863), hsa-miR-210 (ID: 4373089), hsa-miR-7-1 (ID: 001388), hsa-miR-320b (ID: 002844) and hsa-miR-93 (ID: 4373302) at 45°C for 3 hours with the Concentrator 5301 (Eppendorf, Wesseling, Germany). The RT primer pool was re-suspended in 320 µl nuclease-free water. For the validation of miR-1233 in a second cohort, the same RT primer pool was used. Quantitative real-time PCR was performed using the TaqMan Small RNA Assay on the ABIPrism 7900 HT in triplicates in 10 µl reaction volume; all experiments were performed as specified in the manufacturers' protocols. The detailed experimental procedures are presented MIQE-compliant in Methods S1.

### Ethics

All patients gave written informed consent prior blood withdrawal. The study was approved by the Ethikkommission an der Medizinischen Fakultät der Rheinischen Friedrich-Wilhelms-Universität Bonn (approval number: 166/10).

### Statistical methods

The analysis of the real-time PCR data was done using with the SDS software v2.4 (settings: automatic baseline, threshold 0.2); relative microRNA levels were calculated with the RQ Manager v1.2.1, and data was analyzed with DataAssist v2.0 (all software packages: Applied Biosystems). MicroRNA levels in serum were normalized against miR-39 because circulating levels of potential microRNA reference genes are also increased in serum of cancer patients [7] and normalization to a spiked-in synthetic microRNA allows to control for RNA isolation efficiency; tissue microRNA expression was normalized against RNU6, RNU44 and RNU48.

Statistical analyses were performed using PASW statistics 17.0 (SPSS, Chicago, IL, USA): Sensitivity, specificity and area under curve (AUC) for microRNA levels were determined using Receiver Operator Characteristic (ROC) analysis. Clinical-pathological parameters and microRNA levels were correlated using the Mann-Whitney-U or Kruskal-Wallis-test, as appropriate.

## Results

### Phase-1: Marker-Discovery

First, we determined microRNA expression in ccRCC and adjacent non-malignant tissue (both n = 6) of 754 microRNAs using TaqMan Low Density Arrays. MicroRNA expression was normalized to small nuclear RNA (RNU6B, RNU44, RNU48), and the mean expression level was calculated. Eighty-three microRNAs were >2-fold upregulated, and 103 microRNAs were downregulated (<2-fold); similar expression levels were seen in 277 microRNAs and 299 microRNAs were not detectable. See Table S1 for Ct-values.

Next, we determined the microRNA profile in serum from these patients, and compared the expression profile to healthy individuals (each n = 6); in order to control the isolation efficiency, the microRNA expression was normalized to miR-39, which was spiked to serum prior the RNA isolation procedure. In RCC patients, 189 microRNAs were upregulated, 9 downregulated and 120 expressed at similar levels; 444 microRNAs were not detected in serum. Raw data is provided in the Table S1.

**Figure 2:**
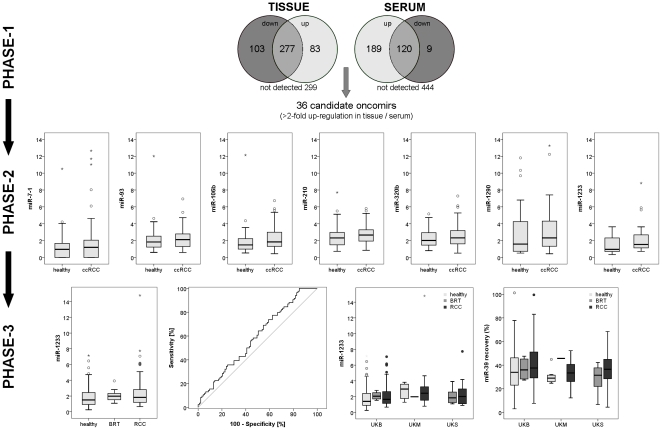
Serum microRNA levels in patients with renal cell carcinoma, benign renal tumors and healthy controls. MicroRNAs were profiled in serum and tissue of patients with clear cell renal cell carcinoma (ccRCC) and healthy controls (each n = 6) using the TaqMan Low Density Array; 36 microRNAs were upregulated in RCC tissue and serum of RCC patients (Phase-1). Seven microRNAs were chosen for verification using real-time PCR in a cohort of 33 ccRCC and 30 healthy individuals (Phase-2). In Phase-3, the finding of increased serum miR-1233 levels in RCC patients was confirmed in a multicentre cohort of 84 RCC, 93 healthy and 13 benign renal tumors (BRT). MicroRNA recovery (as determined using quantification of the synthetic miR-39) was similar in the study centers (UKB, UKM, UKS); the level of miR-1233 were somewhat higher in patients from UKM.

We then compared microRNA profiles of serum and tissue to discover potential tumor-specific microRNAs which could serve as non-invasive diagnostic biomarker: 36 microRNAs were circulating at increased levels in cancer patients and overexpressed in corresponding ccRCC tissue (see Figure 2 and Table 2). The criteria for further investigation of the seven most promising candidates were: *(i)* high microRNA level in serum of cancer patients, *(ii)* high microRNA level in RCC tissue, and *(iii)* quantification cycle values <30 to enable reliable detection. Based on these criteria, we selected miR-106b*, miR-1233, miR-1290, miR-210, miR-7-1*, miR-320b and miR-93.

**Table 2:** MicroRNAs with >2-fold overexpression in serum and tissue of renal cell carcinoma patients.

microRNA	Assay-ID	expression tissue	expression serum
miR-106b	002380	2.708	11.784
miR-1233	002768	2.021	10.600
miR-1290	002863	2.811	6.984
miR-30d*	002305	6.209	5.746
miR-210	4373089	20.718	5.685
miR-425*	002302	3.963	5.091
miR-7-1	001338	2.487	5.051
miR-190b	002263	2.066	4.769
miR-30b	002984	2.103	4.526
miR-93	4373302	2.488	4.429
miR-374a	002125	4.304	4.385
miR-223	002098	2.486	3.972
miR-15a	4373123	3.438	3.927
miR-223	4395406	3.008	3.774
miR-942	002187	2.450	3.591
miR-590-5p	4395176	2.239	3.542
miR-642	4380995	4.241	3.260
miR-193	4395392	2.417	3.257
miR-635*	002432	2.193	3.023
miR-19a	4373099	2.004	2.864
miR-155	4395459	12.898	2.860
miR-16	4373121	2.128	2.813
miR-629*	001562	2.814	2.796
miR-34b	002102	2.556	2.720
miR-301b	4395503	2.765	2.534
miR-1271	002779	3.305	2.504
miR-130b	4373144	2.060	2.478
miR-195	4373105	2.885	2.413
miR-181a	00516	2.563	2.386
miR-21	4373090	4.552	2.355
miR-142-5p	4395359	8.588	2.239
miR-629	4395547	2.972	2.186
miR-25	4373071	2.346	2.176
miR-16-1*	002420	2.360	2.061
miR-15b	4373122	3.318	2.034
miR-23a	4373074	2.128	2.017

### Phase-2: Marker-Verification

The levels of seven promising microRNAs were determined using quantitative real-time PCR in a cohort of 33 patients with ccRCC and 30 urological patients with non-malignant disease; microRNA levels were normalized to miR-39. The level of miR-1233 was significantly increased in patients with RCC (mean 2.19; 95%CI 1.57-2.81) compared to healthy controls (mean 1.49; 95%CI 1.11-1.86; p = 0.022). Using ROC analysis we determined a sensitivity of 90.9% and a specificity of 50% (threshold 0.93; AUC = 0.669, 95%CI = 0.531-0.807). The circulating levels of miR-106b* (p = 0.137), miR-1290 (p = 0.262), miR-210 (p = 0.342), miR-320b (p = 0.308), miR-7-1* (p = 0.199) and miR-93 (p = 0.483) were not different in RCC patients and healthy individuals. See Figure 2.

### Phase-3: Marker-Validation

We thus investigated miR-1233 levels using the same method in an independent cohort of 84 patients with RCC (ccRCC n = 69; pRCC n = 10; chRCC n = 3; sRCC n = 2) and 93 healthy controls; we also analyzed 3 patients with angiomyolipoma and 10 patients with oncocytoma. We could confirm that miR-1233 levels are increased in patients with RCC (p = 0.044): the mean (95% confidence interval) level of miR-1233 was 2.05 (1.58–2.53) in RCC patients and 1.88 (1.61–2.17) in healthy controls. Using ROC analysis we determined a sensitivity of 77.4% and a specificity of 37.6% at a threshold of 1.15 (AUC 0.588, 95% confidence interval 0.505–0.671; accuracy 55.9%; precision 52.4%; false positive rate 63.4%; see Figure 2); sensitivity (84.5%) and specificity (28.5%) were somewhat lower if the threshold of 0.93 was applied, which was determined in the smaller cohort from Phase-1. The diagnostic information was similar if only patients with ccRCC and healthy controls were compared (AUC 0.590, 95% confidence interval 0.503–0.678; accuracy 53.1%; precision 46.8%; false positive rate 63.4%). It should also be noted that miR-1233 levels from RCC patients and healthy controls at the UKM were somewhat increased compared to patients from UKB or UKS (see Figure 2). However, the number of samples from the centers differed (UKB: 85 healthy, 4 BRT, 56 RCC; UKM: 8 healthy, 1 BRT, 14 RCC; UKS: 8 BRT, 14 RCC), and make more detailed interpretation difficult.

Due to the low number of patients with pRCC, chRCC or sRCC, we did not compare miR-1233 levels within different histological subtypes. Clinical-pathological parameters were not correlated with miR-1233 levels in RCC patients (sex: p = 0.300; pT-stage: p = 0.735; lymph node metastasis: p = 0.715; Fuhrman grade: p = 0.215). Of interest, patients with benign renal tumors (angiomyolipoma, oncocytoma) had similar miR-1233 levels as cancer patients (see Figure 2).

In an earlier study [7], we reported lower microRNA isolation efficiencies in patients with prostate cancer; in the present study, patients with RCC had a similar recovery of miR-39: mean (95% CI) recovery was 37.8% (34.2%–41.4%) in RCC patients and 36.1% (32.5%–39.7%) in healthy controls. However, microRNA isolation was quite variable with a standard deviation of 16.7%. We did not notice a difference of microRNA recovery between the study centers.

## Discussion

Pioneering studies on circulating serum/plasma microRNAs [4]–[6] showed that circulating microRNAs are potential tumor markers: microRNAs are stable in blood and their signatures are independent of age and sex. Consequently, researchers demonstrated increased microRNA levels in serum/plasma of patients with various tumor entities [7]–[9]; [11]–[15]. Former studies demonstrated that microRNA expression in RCC and non-malignant renal tissue is different [16]–[24], although findings regarding the number and type of up-/down-regulated microRNAs are conflicting. Thus, circulating microRNAs may also be a suitable biomarker for patients with RCC. To the best of our knowledge, this is the first study on circulating microRNAs in patients with RCC.

So far, there is no consensus on a set of upregulated microRNAs in RCC which could be used as non-invasive biomarker. We therefore compared the microRNA expression profile of normal and cancerous serum/tissue; this approach allowed the identification of 36 microRNAs with relative tumor-specificity upregulated in serum of RCC. Most of these microRNAs were already recognized as overexpressed in RCC tissue in previous studies (e.g. miR-15a, miR-16, miR-21, miR-34b, miR-106b, miR-155, miR-210, miR-142-5p [16]–[25]), whereas miR-1290 and miR-30d were downregulated [25]. The accordance of upregulated microRNAs in our study with earlier reports indicates the validity of our findings in Phase-1, even though the discovery cohort in our study was restricted to each six RCC and control tissue/serum samples. It is of great interest that our study is the first to identify miR-1233 as RCC-associated oncomir. Similar to earlier studies, we observed that microRNAs were predominantly downregulated (103 vs. 83) in RCC tissue. In comparison, an overwhelming number of microRNAs were increased in serum of RCC patients (189 vs. 9). This indicates that circulating microRNAs are generally increased in RCC patients, and that, besides a limited amount of more tumor-specific microRNAs, many of these upregulated serum microRNAs originate from healthy cells. We earlier noted similar findings for cell-free serum DNA, which is mainly composed of DNA from normal cells [26]–[29], and hypothesized that pro-apoptotic signals from cancer-cells lead to cell death of adjacent normal cells. However, additional reasons for this difference are possible: (*i*) In order to identify cancer-associated microRNAs in serum, we compared microRNA expression profiles of serum, cancerous and non-malignant tissue of the same cancer patient, whereas serum from different individuals was used. (*ii*) The different normalization strategy in serum (cel-miR-39) and tissue (RNU6, RNU44, RNU48) may also contribute to different numbers of upregulated microRNAs in tissue and serum.

We next validated the diagnostic potential of seven candidates from the array experiments in cohort of ccRCC patients. Surprisingly, only miR-1233 was circulating at increased concentrations in patients with RCC, whereas differences were no longer observed for miR-106b*, miR-1290, miR-210, miR-320b, miR-7-1* and miR-93. Importantly, we were able to confirm the increase of miR-1233 in serum of RCC patients compared to healthy controls in a multicentre-cohort. Although the diagnostic information (sensitivity 77%, specificity 37.6%, AUC 0.588) was below the expectations after the initial experiments, it should be kept in mind that “accepted” diagnostic biomarkers like PSA do not have a better diagnostic performance for prostate cancer. A relatively small number (n = 13) of oncocytoma and angiomyolipoma did not show different miR-1233 levels, and thus the value of miR-1233 for the differential diagnosis of solid renal tumors needs to be clarified in larger-scaled, future studies. A recent study published by Youssef et al. [24] demonstrated that microRNA expression profiles allow to distinguish between RCC and oncocytoma, thus maybe more than a single microRNA needs to be investigated to improve the diagnostic information.

Our study is the first on miR-1233, and information about the function of this microRNA located on chromosome 15q14 is missing. The microRNA target prediction software miRWalk [30] identified several potential microRNA binding sites: Most of them are genes with unknown function, but there are also tumor-associated genes like BLCAP (identified as tumor suppressor gene in bladder cancer [31]) or p53 among the potential targets.

A strength of our study is the use of samples from three different study centers for validation; other studies on circulating microRNAs in cancer patients only analyzed samples from one centre. Interestingly, we observed higher levels of miR-1233 in healthy individuals and cancer patients in one centre (UKM). This finding must be interpreted with caution because of the different number of patients recruited in each centre, but indicates clearly the necessity of multicentre evaluation of novel biomarkers.

We decided to normalize the amount of circulating microRNAs against cel-miR-39 (as earlier described by Mitchell et al. [4]), which allows absolute quantification of circulating microRNAs with normalization to technical variability. Often, larger RNA species (i.e. U6 or RNU6b) are prone to degradation by serum RNases, and thus normalization to these classical reference genes for microRNA studies is not reasonable. An effective normalization strategy for biological variation is not well-developed and factors leading to biological variation independent of RCC are not known, and their effect on specific microRNAs has not been characterized [32]. Thus, absolute quantification is an appropriate approach for the analysis of circulating microRNAs.

### Limitations

Some limitations should be named: The number of samples in Phase-1 was limited to six, and more samples would have probably allowed us to identify a better microRNA panel for verification/validation. Also, samples from six healthy individuals who were younger than the RCC patients served as controls, and these samples could be a potential bias. In addition, the cancer cohort in phase-1 consisted only of patients with ccRCC, and is therefore less suitable to identify a marker for patients with non-ccRCC histology. Larger-scaled future studies are warranted to explore the role of circulating microRNAs in RCC patients; it is of outstanding importance to analyze serum of patients with non-ccRCC and non-malignant renal tumors for a better understanding of microRNAs is biomarkers for RCC. However, we were able to identify miR-1233 as cancer-associated, cell-free circulating microRNA with potential use in the diagnosis of RCC in the multicenter validation cohort. We did not observe a correlation of miR-1233 and clinical-pathological parameters; it should be kept in mind, that recruiting was performed for a diagnostic study, and thus the composition of the study cohort is unlikely to reveal prognostic information (most patients have moderately differentiated (74% G2) and organ-confined (70%) tumors). Novel biomarkers for RCC should also provide information during follow-up to aid the diagnosis of recurrence; blood samples for this purpose were not available, and thus this question has to be answered by future studies.

## Supporting Information

Table S1 (XLS)

Methods S1 (PDF)
