# Supplementary material for: MicroRNAs in Renal Cell Carcinoma: Diagnostic Implications of Serum miR-1233 Levels
Source: PLoS One. 2011 Sep 30;6(9):e25787. doi: 10.1371/journal.pone.0025787 (PMC3184173; doi:10.1371/journal.pone.0025787)
Supplement: Methods S1 — (PDF) [file pone.0025787.s002.pdf]

EXPERIMENTAL DESIGN

Definition of experimental and control groups

We analyzed separate samples at each step of the study; serum samples were not pooled at any time.

Phase-1: The experimental group consisted of patients undergoing radical nephrectomy or nephron-sparing surgery due to clear cell renal carcinoma (ccRCC). We also investigated a control group consisting of patients with non-malignant disease (men/women attending our hospital for preventive medical examination). Cancerous serum as well as cancerous tissue and non-malignant tissue of the same cancer patient and serum from different healthy individuals were used.

Phase-2 &Phase-3: The experimental group consisted bn of patients undergoing radical nephrectomy or nephron-sparing surgery for renal tumors; thus, the study cohort consisted of patients with renal cell carcinoma and benign renal tumors (BRT; i.e. oncocytoma and angiomyolipoma). We also investigated a control group consisting of patients with non-malignant disease (men/women attending our hospital for surgery of non-malignant disease, i.e. benign prostate enlargement or urinary incontinence, or preventive medical examination). Only serum samples were used.

Number within each group

Phase-1: ccRCC n=6, healthy control n=6.

Phase-2: ccRCC n=33, healthy control n=30.

Phase-3: renal cell carcinoma n=84 (including 69 ccRCC, 10 papillary renal cell carcinoma, 3 chromophobe renal cell carcinoma and 2 sarcomatoid renal cell carcinoma patients), healthy control n=93, angiomyolipoma n=3, oncocytoma n=10. See Table 1 for detailed clinical-pathological parameters.

Assay carried out by core lab or investigator's lab?

All formalin-fixed, paraffin embedded tissue samples used were collected and stored in the archival files at the Institut für Pathologie at the Universitätsklinikum Bonn.

Serum samples derived from three different universities (UKB: 85 healthy, 4 benign renal tumor, 56 renal cell carcinoma; UKM: 8 healthy, 1 benign renal tumor, 14 renal cell carcinoma; UKS: 8 benign renal tumor, 14 renal cell carcinoma).

RNA purification and qPCR of all samples was done at the laboratories of the Klinik und Poliklinik für Urologie und Kinderurologie at the Universitätsklinikum Bonn.

Acknowledgement of authors' contributions

Study concept and design: Wulfken, Müller, Ellinger.

Acquisition of data: Wulfken.

Analysis and interpretation: Wulfken, Ellinger.

Drafting the manuscript: Wulfken, Ellinger.

Critical revision of the manuscript for important intellectual content: Moritz, Ohlmann, Holdenrieder, Jung, Becker, Herrmann, Walgenbach-Brünagel, von Ruecker, Müller

Statistical analysis: Wulfken, Ellinger.

Obtaining funding: Ellinger (Deutsche Forschungsgemeinschaft, EL-623/1-1).

Administrative, technical, or material support: Holdenrieder, Walgenbach-Brünagel, Müller, von Ruecker.

Supervision: von Ruecker, Müller, Ellinger.

SAMPLE

Description
Phase-1: We used formalin-fixed, paraffin-embedded tissues (renal cell carcinoma and adjacent to cancer located normal tissue) and serum samples (withdrawn prior to surgery).
Phase-2 & Phase-3: Serum samples, withdrawn prior to surgery, were used.

Volume/mass of sample processed
Tissue samples: Five serial sections (20 µm) were cut from the tissue block and used for RNA purification.
Serum samples: A total of 400 µl serum was used for RNA purification.

Microdissection or macrodissection
After being cut from the tissue block, tissue was dissected using a scalpel to enrich ccRCC and adjacent non-malignant renal tissue according to the haematoxylin and eosin stained section.

Processing procedure
Tissue samples: Tissue samples were collected, fixed in formalin and embedded in paraffin, and stored within the archival files of the Institut für Pathologie at the Universitätsklinikum Bonn. The RNA was purified immediately after macrodissection.
Serum samples: The collection of serum samples was performed according to the SOPs of the Biobank at the CIO Köln/Bonn: Serum samples were withdrawn prior surgery and stored at -80℃. A total of 400 µl seru m was used for RNA purification.

If frozen - how and how quickly?
Blood samples were withdrawn prior to surgery; after clotting, serum was separated after centrifugation within 60-180 minutes and stored in cryotubes at -80℃ until use.

If fixed - with what, how quickly?
Tissue samples were formalin-fixed immediately after surgery and transferred to the Institut für Pathologie. Paraffin embedding was performed after a 24 hour fixation.

Sample storage conditions and duration (especially for FFPE samples)
Formalin-fixed, paraffin embedded tissues, collected between 2006 and 2010, were stored in the archival files of the Institut für Pathologie at the Universitätsklinikum Bonn. Serum samples, collected between 2005 and 2011, were stored in cryotubes at -80℃.

NUCLEIC ACID EXTRACTION

Procedure and/or instrumentation
RNA isolation was performed according to the manufacturer’s recommendations using the mirVana PARIS Kit (catalog number AM-1556; Ambion, Foster City, CA, USA) and the RecoverAll Total Nucleic Acid Isolation Kit (catalog number AM-1975; Ambion, Foster City, CA, USA) (for modifications see next point).

Name of kit and details of any modifications
Tissue samples: RNA was isolated with the RecoverAll Total Nucleic Acid Isolation Kit (Ambion, Foster City, CA, USA) according to the manufacturer´s

recommendations (final elution volume 50 µl) with one exception: after having air dried the tissue pellet for 120 minutes, we incubated the sample in heat blocks at 50℃ for 30 minutes.
Serum samples: The mirVana PARIS Kit (Ambion, Foster City, CA, USA) was used to purify total RNA from 400µl serum. RNA isolation was performed according to the manufacturer’s recommendation (final elution volume 50 µl) with one exception: we added 25 fmol of a synthetic Caenorhabditis elegans microRNA, cel-miR-39 (Qiagen, Hilden, Germany; catalog number MSY0000010) to the serum at the beginning of the isolation procedure.

Source of additional reagents used
Synthetic Caenorhabditis elegans microRNA, cel-miR-39 (Qiagen, Hilden, Germany; catalog number MSY0000010). 2-Mercaptoethanol (Sigma, St. Louis, MO, USA). 100% Ethanol (Merck, Darmstadt, Germany; index number 603-002-00-5)

Details of DNase or RNAse treatment
DNase treatment was performed according to the manufacturer’s recommendation in tissue samples; we did not perform DNase treatment for serum samples.

Contamination assessment (DNA or RNA)
RNA from tissue samples was analyzed on a Nanodrop 1000 spectrophotometer (ThermoScientific, Wilmington, DE; USA) to assess the RNA concentration and purity.

Purity (A260/A280)
The ratio A260/A280 was 1.80 to 2.05 in the tissue samples. Due to the low amount of RNA obtained from serum samples, we did not perform Nanodrop measurements for these samples.

RNA integrity method/instrument
RNA integrity was not checked for the following reasons: (*i*) microRNAs are unlikely being degraded in formalin-fixed paraffin embedded tissues due to their small size. (*ii*) The amount of isolated RNA in serum was too small for RNA integrity analysis.

RIN/RQI or Cq of 3' and 5' transcripts
RNA integrity was not checked.

Electrophoresis traces
RNA integrity was not checked.

Inhibition testing (Cq dilutions, spike or other)
The addition cel-miR-39 (Qiagen, Hilden, Germany; catalog number MSY0000010) to the serum and its quantification allowed controlling technical variability.

REVERSE TRANSCRIPTION

Complete reaction conditions
Phase-1: The TaqMan MicroRNA Reverse Transcription Kit (catalog number: 4366596; Applied Biosystems, Foster City, CA, USA), Megaplex RT Primer Pools A v2.1 (catalogue number: 4399966; Applied Biosystems, Foster City, CA, USA) and Megaplex RT Primer Pools B v3.0 (catalog number: 4399966; Applied Biosystems,

Foster City, CA, USA) were used to synthesize single-stranded cDNA from total RNA samples. All reactions were performed as specified in the protocols of the manufacturer: 3 µl total RNA were added to 4.5 µl of the RT reaction mix (Megaplex RT Primers 10X, dNTPs with dTTP 100mM, MultiScribe Reverse Transcriptase 50U/µl, 10X RT Buffer, $MgCl_2$ 25 mM, RNase Inhibitor 20 U/µl and Nuclease-free water). After incubation on ice for 5 minutes reverse transcription was performed using a thermal cycler (UNO-Thermoblock, Biometra, Göttingen, Germany).
In order to increase the sensitivity of the TaqMan Low Density Arrays, we performed a pre-amplification after the reverse transcription using the TaqMan PreAmp Mastermix (catalog number: 4384267; Applied Biosystems, Foster City, CA, USA) as well as the Megaplex PreAmp Primer Pools A v2.1 (catalog number: 4399233; Applied Biosystems, Foster City, CA, USA) and Megaplex PreAmp Primer Pools B v3.0 (catalog number: 4399201; Applied Biosystems, Foster City, CA, USA). All reactions were performed as specified in the protocols of the manufacturer.
Phase-2 & Phase-3: The TaqMan MicroRNA Reverse Transcription Kit and specific RT primers were used to synthesize single-stranded cDNA from total RNA samples. All reactions were performed as specified in the protocols of the manufacturer with one exception: Reverse Transcription was performed with a self-created primer pool. To obtain this RT primer pool (20X), we speed vacuumed a mixture of each 320 µl of cel-miR-39 (Applied Biosystems Assay ID: 000200), hsa-miR-1233 (ID: 002768), hsa-miR-106b* (ID: 003280), hsa-miR-1290 (ID: 002863), hsa-miR-210 (ID: 4373089), hsa-miR-7-1 (ID: 001388), hsa-miR-320b (ID: 002844) and hsa-miR-93 (ID: 4373302) at 45°C for 3 hours with the Concentr ator 5301 (Eppendorf, Wesseling, Germany). The RT primer pool was re-suspended in 320µl nuclease-free water. Then 5 µl total RNA were added to 10 µl of the RT reaction mix (RT primer pool 20X, dNTPs with dTTP 100 mM, MultiScribe Reverse Transcriptase 50 U/µl, 10X Reverse Transcription Buffer, RNase Inhibitor 20 U/µl and Nuclease-free water). After incubation on ice for 5 minutes, reverse transcription was performed on a thermal cycler (UNO-Thermoblock, Biometra, Göttingen, Germany).

Amount of RNA and reaction volume
Phase-1: We used 3 µl of isolated serum RNA and 16.667 ng/µl tissue RNA for reverse transcription. The reaction volume for all samples was 7.5 µl. For pre-amplification 2.5 µl RT product were used and the reaction volume was 25 µl.
Phase-2 & Phase-3: We used 5 µl of isolated RNA from serum for cDNA synthesis. The reaction volume was 15 µl.

Priming oligonucleotide (if using GSP) and concentration
Phase-1: We used stem-looped RT primers purchased from Applied Biosystems (RT Primer Pools A v2.1: catalog number: 4401009; and RT Primer Pools B v3.0: catalog number: 4401010; Foster City, CA, USA) according to the manufacturer´s protocol. For pre-amplification we used the Megaplex PreAmp Primers purchased from Applied Biosystems (Megaplex PreAmp Primer Pools A v2.1: catalog number: 4399233; and Megaplex PreAmp Primer Pools B v3.0, catalog number: 4399201; Foster City, CA, USA).
Phase-2 & Phase-3: We used stem-loop RT primers purchased from Applied Biosystems (Foster City, CA, USA). Reverse transcription was performed with a self-created primer pool. To obtain this RT primer pool (20X), we speed vacuumed a mixture of each 320 µl of cel-miR-39 (Applied Biosystems Assay ID: 000200), hsa-miR-1233 (ID: 002768), hsa-miR-106b* (ID: 003280), hsa-miR-1290 (ID: 002863), hsa-miR-210 (ID: 4373089), hsa-miR-7-1 (ID: 001388), hsa-miR-320b (ID: 002844)

and hsa-miR-93 (ID: 4373302) at 45℃ for 3 hours with the Concentrator 5301 (Eppendorf, Wesseling, Germany). The RT primer pool was re-suspended in 320µl nuclease-free water.

Reverse transcriptase and concentration
Phase-1: MultiScribe Reverse Transcriptase (50 U/µl) (Applied Biosystems, Foster City, CA, USA), component of the TaqMan MicroRNA Reverse Transcription Kit (catalog number: 4366596; Applied Biosystems, Foster City, CA, USA).
Phase-2 & Phase-3: MultiScribe Reverse Transcriptase (50 U/µl) (Applied Biosystems, Foster City, CA, USA), component of the TaqMan MicroRNA Reverse Transcription Kit (catalog number: 4366596; Applied Biosystems, Foster City, CA, USA).

Temperature and time
Phase-1: 40 cycles: with 2 minutes at 16 ℃ , 1 minute at 42 ℃, 1 second at 50 ℃ and 5 minutes 85 ℃; rest period at 4℃. The preamplification reaction was performed as specified in the manufacturer`s protocol: 10 minutes at 95℃, 2 minutes at 55℃, 2 minutes at 72℃, followed by 12 cycles with 15 seconds at 95℃ and 4 minutes at 60℃, finally 10 minutes at 99.9 ℃; rest period at 4℃.
Phase-2 & Phase-3: 30 minutes at 16℃ followed by 30 minutes at 42℃ and 5 minutes at 85℃; rest period at 4℃.

Manufacturer of reagents and catalogue numbers
Phase-1: TaqMan MicroRNA Reverse Transcription Kit (catalog number: 4366596; Applied Biosystems, Foster City, CA, USA); Megaplex RT Primer Human Pool A v2.1 (catalog number: 4399966; Applied Biosystems, Foster City, CA, USA); Megaplex RT Primer Human Pool B v3.0 (catalog number: 4399968; Applied Biosystems, Foster City, CA, USA).
Preamplification reaction: TaqMan PreAmp Master Mix 2X (catalog number: 4391128; Applied Biosystems, Foster City, CA, USA); Megaplex PreAmp Primer Human Pool A v2.1 (catalog number: 4399233; Applied Biosystems, Foster City, CA, USA); Megaplex PreAmp Primer Human Pool B v3.0 (catalog number: 4399201; Applied Biosystems, Foster City, CA, USA).
Phase-2 & Phase-3: TaqMan MicroRNA Reverse Transcription Kit (catalog number: 4366596; Applied Biosystems, Foster City, CA, USA), RT Primer: cel-miR-39 (assay ID: 000200; Applied Biosystems, Foster City, CA, USA), hsa-miR-1233 (ID: 002768, Applied Biosystems, Foster City, CA, USA), hsa-miR-106b* (ID: 003280; Applied Biosystems, Foster City, CA, USA), hsa-miR-1290 (ID: 002863, Applied Biosystems, Foster City, CA, USA), hsa-miR-210 (ID: 4373089, Applied Biosystems, Foster City, CA, USA), hsa-miR-7-1 (ID: 001388, Applied Biosystems, Foster City, CA, USA), hsa-miR-320b (ID: 002844, Applied Biosystems, Foster City, CA, USA) and hsa-miR-93 (ID: 4373302, Applied Biosystems, Foster City, CA, USA).

Cqs with and without RT
Not determined.

Storage conditions of cDNA
Phase-1: We stored cDNA at -20 ℃ for one week.
Phase-2 & Phase-3: We stored cDNA at -20 ℃ for up to 3 weeks.

qPCR TARGET INFORMATION

If multiplex, efficiency and LOD (=limit of detection) of each assay.
The Low Density Arrays in Phase-1 were purchased pre-designed from Applied Biosystems; efficiency (90-110%) and limit of detection (10 copies) are guaranteed by the manufacturer. We did not perform multiplex PCR in Phase-2 and Phase-3.

Gene symbol
The detailed assay list is provided in the Supplementary Table 1.

Sequence accession number
The detailed assay list is provided in the Supplementary Table 1.

Location of amplicon
All primers were purchased pre-designed from Applied Biosystems, and the location of the amplicon is not provided by the manufacturer. The assay ID is provided in the Supplementary Table 1.

Amplicon length
All primers were purchased pre-designed from Applied Biosystems, and the size of the amplicon is not provided by the manufacturer. The assay ID is provided in the Supplementary Table 1.

In silico specificity screen (BLAST, etc)
All primers were purchased pre-designed from Applied Biosystems, BLAST searches were performed by the manufacturer. The assay ID is provided in the Supplementary Table 1.

Pseudogenes, retropseudogenes or other homologs?
All primers were purchased pre-designed from Applied Biosystems. The primers do not target pseudogenes, retropseudogenes or other homologs according to the manufacturer. The assay ID is provided in the Supplementary Table 1.

Sequence alignment
All primers were purchased pre-designed from Applied Biosystems. Sequence alignment for each primer (the assay ID is provided in the Supplementary Table 1) is possible at the manufacturer’s website (https://products.appliedbiosystems.com).

Secondary structure analysis of amplicon
All primers were purchased pre-designed from Applied Biosystems, and details on the amplicon are not provided.

Location of each primer by exon or intron (if applicable)
All primers were purchased pre-designed from Applied Biosystems, and details on the location are not provided.

What splice variants are targeted?
All primers were purchased pre-designed from Applied Biosystems; details about the primers are reported in Supplementary Table 1.

qPCR OLIGONUCLEOTIDES

Primer sequences
All primers were purchased pre-designed from Applied Biosystems (see Supplementary Table 1 for the assay IDs). The primer sequence is not supplied by the manufacturer.

RTPrimerDB Identification Number
All primers were purchased pre-designed from Applied Biosystems (see Supplementary Table 1 for the assay IDs). The primers are not listed in the RTPrimer database.

Probe sequences
The TaqMan MGB probe contains: a reporter dye (FAM dye), a minor groove binder MGB and a non-fluorescent quencher (NFQ).

Location and identity of any modifications
All primers were purchased pre-designed from Applied Biosystems, and this information is not provided.

Manufacturer of oligonucleotides
Applied Biosystems, Foster City, CA, USA.

Purification method
All primers were purchased pre-designed from Applied Biosystems, and information on purification is not provided.

qPCR PROTOCOL

Complete reaction conditions
Phase-1: MicroRNA profiling of 754 different human microRNAs was performed using the TaqMan Low Density Array (TaqMan Array Human MicroRNA A+B Cards Set v3.0; see Supplementary Table 1 for a list of microRNAs) on an ABIPrism 7900HT.
Phase-2 & Phase-3: Quantitative real-time PCR was performed using the TaqMan Small RNA Assay on the ABIPrism 7900HT in triplicates (10 µl reaction volume). All experiments were performed as specified in the manufacturer’s protocols.

Reaction volume and amount of cDNA/DNA
Our aim was to identify microRNA levels in serum. As specified in the manufacturer`s protocol we used 3 µl of isolated RNA in Phase-1 and 15 µl of isolated RNA in Phase-2 and Phase-3 for cDNA synthesis. For the analysis of tissue microRNA levels we used 16.667 ng/µl RNA. cDNA concentrations were not determined later since there is no sense in equalizing cDNA concentrations in this study.

Primer, (probe), Mg++ and dNTP concentrations
Phase 1: We used the TaqMan Universal PCR Master Mix No AmpErase UNG (catalog number: 4324018; Applied Biosystems, Foster City, CA, USA) according to the manufacturer`s protocol. The concentration of the primers, Mg++ and dNTPs is not provided by the manufacturer.
Phase 2 & Phase 3: We used the TaqMan Universal PCR Master Mix II without UNG (catalog number: 4440043; Applied Biosystems, Foster City, CA, USA) according to

the manufacturer`s protocol. The concentration of Mg++ and dNTPs is not provided by the manufacturer. The concentration of the primers used was 20X.

Polymerase identity and concentration
Phase 1: We used the TaqMan Universal PCR Master Mix No AmpErase UNG (catalog number: 4324018; Applied Biosystems, Foster City, CA, USA). It includes the AmpliTaq Gold DNA Polymerase. Its concentration is not provided by the manufacturer.
Phase 2 & Phase 3: We used the TaqMan Universal PCR Master Mix II without UNG (catalog number: 4440043; Applied Biosystems, Foster City, CA, USA). It includes the AmpliTaq Gold DNA Polymerase. Its concentration is not provided by the manufacturer.

Buffer/kit identity and manufacturer
Phase 1: We used the TaqMan Universal PCR Master Mix No AmpErase UNG (catalog number: 4324018; Applied Biosystems, Foster City, CA, USA).
Phase 2 & Phase 3: We used the TaqMan Universal PCR Master Mix II without UNG (catalog number: 4440043; Applied Biosystems, Foster City, CA, USA).

Exact chemical constitution of the buffer
Phase 1: We used the TaqMan Universal PCR Master Mix No AmpErase UNG (catalog number: 4324018; Applied Biosystems, Foster City, CA, USA). The exact chemical constitution of the buffer is not provided by the manufacturer.
Phase 2 & Phase 3: We used the TaqMan Universal PCR Master Mix II without UNG (catalog number: 4440043; Applied Biosystems, Foster City, CA, USA). The exact chemical constitution of the buffer is not provided by the manufacturer.

Additives (SYBR Green I, DMSO, etc.)
We did not use any additives.

Manufacturer of plates/tubes and catalog number
For qPCR the following plates and seals were used: Thermo-Fast 384 (catalog number: TF-0384; ThermoScientific, Wilmington, DE; USA) and Absolute QPCR-Seal (catalog number: AB-1170; ThermoScientific, Wilmington, DE; USA).

Complete thermocycling parameters
Phase-1: The thermo cycling conditions were, as specified in the manufacturer's protocol: 2 minutes at 50℃ and 10 minutes at 94.5° C, followed by 40 cycles with 30 seconds at 97℃ and 1 minute at 59.7℃.
Phase-2 & Phase-3: The thermo cycling conditions were, as specified in the manufacturer's protocol: 10 minutes at 95℃, follow ed by 40 cycles with 15 seconds at 95℃ and 60 seconds at 60℃.

Reaction setup (manual/robotic)
The qPCR was setup up manually using an 8-chanel pipette (Matrix 30 µl, ThermoScientific, Wilmington, DE; USA).

Manufacturer of qPCR instrument
ABIPrism 7900HT (Applied Biosystems, Foster City, CA, USA).

qPCR VALIDATION

Evidence of optimisation (from gradients)
All primers were purchased pre-designed from Applied Biosystems, and performance is guaranteed by the manufacturer.

Specificity (gel, sequence, melt, or digest)
All primers were purchased pre-designed from Applied Biosystems, and performance is guaranteed by the manufacturer.

For SYBR Green I, Cq of the NTC
Not applicable.

Standard curves with slope and y-intercept
All primers were purchased pre-designed from Applied Biosystems, and performance is guaranteed by the manufacturer.

PCR efficiency calculated from slope
All primers were purchased pre-designed from Applied Biosystems, and performance is guaranteed by the manufacturer.

Confidence interval for PCR efficiency or standard error
All primers were purchased pre-designed from Applied Biosystems, and performance is guaranteed by the manufacturer.

r^2 of standard curve
All primers were purchased pre-designed from Applied Biosystems, and performance is guaranteed by the manufacturer.

Linear dynamic range
All primers were purchased pre-designed from Applied Biosystems, and performance is guaranteed by the manufacturer.

Cq variation at LOD
All primers were purchased pre-designed from Applied Biosystems, and performance is guaranteed by the manufacturer.

Confidence intervals throughout range
All primers were purchased pre-designed from Applied Biosystems, and performance is guaranteed by the manufacturer.

Evidence for limit of detection (LOD)
All primers were purchased pre-designed from Applied Biosystems, and performance is guaranteed by the manufacturer.

If multiplex, efficiency and LOD of each assay.
We did not perform multiplex assays.

DATA ANALYSIS

qPCR analysis program (source, version)
The analysis of the real-time PCR data was done using the SDS software v2.4 (settings: automatic baseline, threshold 0.2); relative microRNA levels were calculated with the RQ Manager v1.2.1, and data was analyzed with DataAssist v2.0 (all software packages: Applied Biosystems, Foster City, CA, USA).

Cq method determination
The relative quantification of microRNA expression was performed using the comparative CT (ΔΔCT) method using the DataAssist v2.0 software.

Outlier identification and disposition
By analyzing the real-time PCR data using the SDS software v2.4 with the following settings *automatic baseline, threshold 0.2*, outliers were identified and erased.

Results of NTCs
NTCs gave no signal.

Justification of number and choice of reference genes
Tissue samples: The reference genes (RNU6B, RNU44 and RNU48) were already spotted on the Low Density Arrays by the manufacturer and used for the analysis of microRNAs in tissue; the use of these reference genes is recommended by Applied Biosystems, and RNU6B, RNU44 and RNU48 are commonly used reference genes.
Serum samples: We normalized the amount of circulating microRNAs against cel-miR-39, which allows absolute quantification of circulating microRNAs with normalization to technical variability. Larger RNA species (i.e. RNU6B) are prone to degradation by serum RNases, and thus normalization to these classical references genes for serum microRNA studies is not reasonable.

Description of normalisation method
Tissue microRNA levels were normalized to RNU6, RNU44 and RNU48 using the comparative CT (ΔΔCT) method; calculations were performed with DataAssist v2.0. Serum microRNA levels were normalized to cel-miR-39 (synthetic non-human-microRNA, spiked-in during RNA isolation) using the comparative CT (ΔΔCT) method; calculations were performed with DataAssist v2.0.

Number and concordance of biological replicates
Not tested.

Number and stage (RT or qPCR) of technical replicates
In Phase-1 Low Density Arrays were performed without technical replicates. All PCR experiments (i.e. Phase-2 and Phase-3) were performed with three technical replicates.

Repeatability (intra-assay variation) and Reproducibility (inter-assay variation)
The variation of the microRNA quantification was tested by the following procedure: eight serum RNA isolates were pooled, and divided into 3 portions, which were separately reverse transcribed and quantified using qPCR (in triplicates). The coefficient of variation was 6.8% (miR-106b), 15.4% (miR-1233-2), 11.5% (miR-1290), 7.6% (miR-210), 11.2% (miR-320b), 17.9% (miR-7-1) and 5.4% (miR-93).

Power analysis
Not done.

Statistical methods for result significance
Sensitivity, specificity and area under curve (AUC) for serum microRNA levels were determined using Receiver Operator Characteristic (ROC) analysis. Clinical-pathological parameters and microRNA levels were correlated using the Mann-Whitney-U or Kruskal-Wallis-test, as appropriate.

Software (source, version)
Statistical analyses were performed using PASW statistics 17.0 (SPSS, Chicago, IL, USA).

Cq or raw data submission using RDML
Cq data from the Low Density Arrays is provided as supplementary file.
